# Genetic Diversity and Phylogenetic Relationships of Annual and Perennial *Glycine* Species

**DOI:** 10.1534/g3.119.400220

**Published:** 2019-05-16

**Authors:** Eun-Young Hwang, He Wei, Steven G. Schroeder, Edward W. Fickus, Charles V. Quigley, Patrick Elia, Susan Araya, Faming Dong, Larissa Costa, Marcio Elias Ferreira, Perry B. Cregan, Qijian Song

**Affiliations:** * Department of Plant Sciences and Landscape Architecture, University of Maryland, College Park, MD 20742; † Institute of Industrial Crops, Henan Academy of Agricultural Sciences, Zhenzhou, Henan Province, 450002, China; ‡ United States Department of Agriculture, Agricultural Research Service,, Bovine Functional Genomics Laboratory, Animal and Natural Resources Institute, Beltsville, MD, 20705; § United States Department of Agriculture, Agricultural Research Service, Soybean Genomics and Improvement Laboratory, Beltsville, MD 20705; ** College of Life Science and Technology, Huazhong Agricultural University, Wuhan, 430070, China; †† EMBRAPA Genetic Resources and Biotechnology, Embrapa, Brasília, DF, C.P.02372, Brazil

**Keywords:** soybean, perennial crop relatives, nucleotide diversity, phylogenetic analysis, divergence, trans-specific polymorphism

## Abstract

We have estimated the average genetic diversity of two *Glycine* annual and six perennial species based upon 76 orthologous gene sets and performed phylogenetic analysis, divergence analysis and tests for departure from neutrality of the eight species using 52 orthologous gene sets. In addition, 367 orthologous gene sets were used to estimate the relationships of 11 *G. canescens* accessions. Among the perennials, *G. canescens* showed the highest nucleotide diversity. The other perennials, except for *G. tomentella*, had higher nucleotide diversity than the two annuals. Phylogenetic analysis of the *Glycine* showed a similar genome grouping with the previous report except for *G. cyrtoloba* and *G. stenophita* which formed a sister clade in the study. Divergence analysis supported the phylogenetic relationships that *G. falcata* was the most divergent from *G. max*, followed by *G. cyrtoloba*, *G. syndetika*, *G. tomentella* D3, *G. stenophita* and *G. canescens*. Most genic sequences were homogeneous in the levels of polymorphism and divergence between *G. max* and other *Glycine* species based on the HKA test, thus, *Glycine* perennials may have experienced a very similar evolution as inferred by *trans*-specific mutation analysis. The greater genetic diversity of most perennial *Glycine* species and their origins from the warmer and drier climates of Australia suggests the perennials maybe a potential source of heat and drought resistance that will be of value in the face of climate change.

The genus *Glycine* Willd. includes two subgenera, *Glycine* and *Soja* (Moench) F.J. Herm. Subgenus *Glycine* contains 25 named and at least six unnamed perennial taxa (González-Orozco *et al.* 2012; Sherman-Broyles *et al.* 2014). Subgenus *Soja* contains two annual species, *Glycine max* (L.) Merr., the cultivated soybean, and *Glycine soja* Sieb. & Zucc., the wild soybean. The two subgenera shared a common ancestor approximately 5 million years ago (Innes *et al.* 2008; Egan and Doyle 2010), however, the biogeography of *Glycine* is unusual in that the annuals are native to northeastern Asia whereas the diploid (2n = 38, 40) perennials are almost exclusively Australian (Doyle *et al.* 2004).

The perennial *Glycine* species have been demonstrated to be potential sources of economically important traits, especially disease resistance genes, for use in cultivated soybean. Approximately 41% of 294 accessions of the 12 perennial *Glycine* species examined had moderate or high resistance to soybean rust (Hartman *et al.* 1992) and *G. canescens* had at least four soybean rust resistance loci (Burdon 1988). In addition, some accessions of most perennial *Glycine* species showed resistance to one of the most destructive soybean pests, the soybean cyst nematode (SCN) (Bauer *et al.* 2007; Riggs *et al.* 1998). These authors also determined that progenies derived from *G. max* (SCN susceptible) x *G. tomentella* (SCN resistant) showed resistance to SCN. These studies verified the perennial *Glycine* as a source of useful genes to improve cultivated soybean, although currently hybridization between annual and perennial *Glycine* species is limited (Ratnaparkhe *et al.* 2011). The other important aspect of the perennial *Glycine* is that some of these species have been adapted to harsh environments, especially very dry and hot areas.

Early studies were focused on the collection of germplasm and the classification of the collection based upon crossability testing, cytogenetic analysis, as well as molecular marker analysis such as isozyme grouping and nucleotide variation in nuclear or chloroplast DNA (Singh and Hymowitz 1985a; Brown *et al.* 1990; Singh *et al.* 1992; Kollipara *et al.* 1994, 1997; Singh *et al.* 1998; Doyle *et al.* 1999a; Doyle *et al.* 2003b; Grant *et al.* 1984; Doyle *et al.* 1999b; Doyle *et al.* 2003a; Ratnaparkhe *et al.* 2011; Sherman-Broyles *et al.* 2014). These efforts established the phylogenetic relationships among the *Glycine* species indicating that the two annuals belong to the G genome group and the 25 perennials (Doyle *et al.* 2004; Sherman-Broyles *et al.* 2014) to the nine genome groups from A to I (Hymowitz *et al.* 1998). However, because of the lack of DNA sequence information for the perennials, relatively little is known about the genetic diversity of the perennial *Glycine* species and the relationships among the perennial species as well as their relationship with cultivated soybean. As these relationships could be an indicator of the successful rate of the crossing and the difference between the perennial species and cultivated soybean, this information could help us to choose perennial species for the improvement of cultivated soybean.

The development of high throughput DNA sequence analysis makes it possible to identify many orthologous gene sets in the *Glycine* species with a high efficiency. In this study, we estimated the average genetic diversity of six perennial *Glycine* species chosen to represent the phylogenetic diversity of subgenus *Glycine* (Ratnaparkhe *et al.* 2011), *G. canescens*, *G. cyrtoloba*, *G. falcata*, *G. stenophita*, *G. syndetika*, and *G. tomentella* D3, and the two annual *Glycine* species, *G. soja* and *G. max*, based upon the DNA sequence analysis of 76 orthologous gene sets. Phylogenetic analysis, evolutionary divergence analysis among the species and test for departure from neutrality, were performed using common sequences of 52 orthologous gene sets with 87.6 Kbp in length in 77 accessions of the *Glycine* species. In addition, approximately 462 Kbp of sequences from 367 orthologous gene sets were used to estimate the relationships of 11 *G. canescens* accessions.

## Materials and Methods

### Plant materials

Both annual *Glycine* species, *G. soja* (G genome) and *G. max* (G genome), were used for this study along with six perennial *Glycine* species, *G. canescens* (A genome), *G. cyrtoloba* (C genome), *G. falcata* (F genome), *G. stenophita* (B’ genome), *G. syndetika* (A genome; previously named *G. tomentella* D4), and *G. tomentella* D3 (D genome). The six perennials were chosen to represent the major clades of subgenus *Glycine* (Sherman-Broyles *et al.* 2014), which are well-correlated with the “genome groups” classified by Singh and Hymowitz (1985a) and Hymowitz *et al.* (1998). There were two sets of accessions of each species used for the current study. The data set used for the estimation of genetic diversity was based on 100 accessions and the data set used for phylogenetic analysis, evolutionary divergence analysis among the species and test for departure from neutrality, included 77 accessions of the eight *Glycine* species. The accessions used for each study are listed in Supplementary File 1. The perennial *Glycine* species are basically inbred, they have similar low out-crossing rates as cultivated soybean. In the greenhouse, the pods from some species were formed even though the flowers did not blossom. In addition, very few heterozygotic single nucleotide polymorphic sites were observed from the sequence of the gene sets within each species.

### Sequence diversity analysis

#### Genomic DNA extraction:

**Figure 1:**
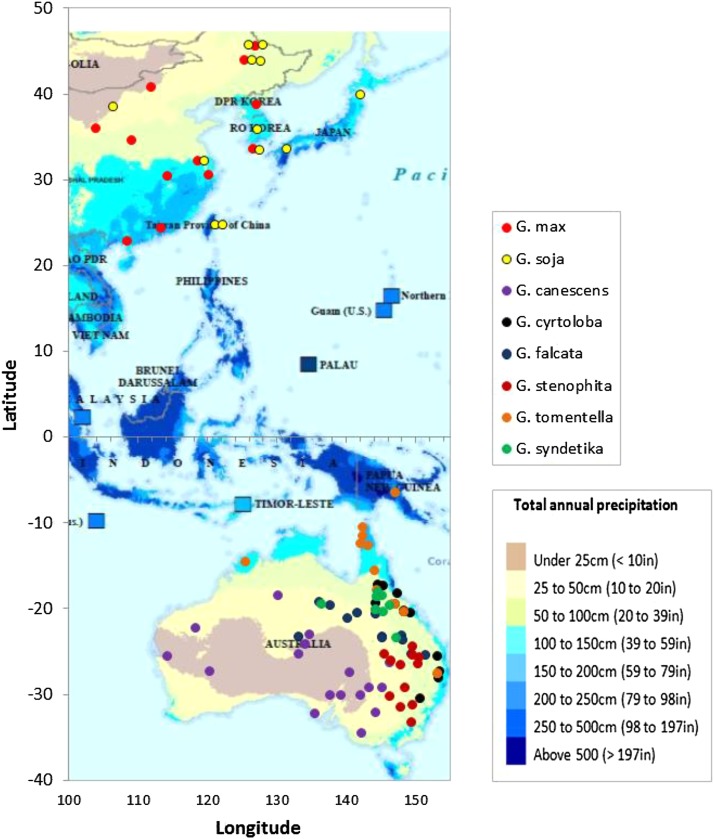
Geographical origin of the accessions. The origin of 98 accessions of the two annual and the six perennial *Glycine* species and the average annual precipitation in the corresponding regions.

Genomic DNA of 10 to 15 accessions of each species (Supplementary File 1) was isolated from young leaves of greenhouse-grown plants using the CTAB method (Keim *et al.* 1988). In an effort to sample the breadth of diversity within each species, all accessions were selected based on geographical locations that covered the entire range over which each species had been collected (Figure 1).

#### RNA extraction and cDNA preparation:

For each of six perennial species a single seed of one accession was scarified and planted in a 1:1 soil and sand mix and grown in the greenhouse. Leaves, pods, and flowers were harvested from single plants and roots were harvested from 5 to 10 seedlings grown on a wet paper towel in a petri-dish for 3 to 7 days. Total RNA was extracted from leaf, pod, flower and root tissue from each of the six perennial species using the Qiagen mini-RNA prep (Qiagen, Valencia, CA). Poly-A^+^ RNA was isolated from the total RNA using a Dynabead mRNA purification kit (Invitrogen, Carlsbad, CA) and cDNA libraries were constructed following the protocol “Preparing samples for sequencing of mRNA” (Illumina Inc., San Diego, CA).

#### Sample preparation for sequence analysis:

The 6 cDNA libraries, constructed from different tissues of the six perennial species, were fragmented using NEB Next dsDNA fragmentase for 20 min (NEB, Beverly, MA), end repaired and an ‘A’ overhang was added to the ends of the fragments. The end repaired cDNA libraries were ligated with the Illumina paired-end sequencing multiplex adapters and run on a 2% agarose gel for size selection. Fragments ranging from 100 to 150 bp and from 450 to 550 bp were isolated and normalized to reduce the relative number of highly expressed genes (Zhulidov *et al.* 2004) using double strand specific nuclease (Evrogen, Moscow, Russia). The normalized cDNA was PCR amplified for 17 or 18 cycles. The concentration of the amplified cDNA was verified using the Agilent 2100 Bioanalyzer (Agilent Technologies, Palo Alto, CA). The cDNAs from different tissues of the same accession were combined to obtain equimolar concentrations of each species and run on the Illumina Genome Analyzer IIx to obtain 105 bp paired-end sequence reads.

#### Sequence analysis and selection of orthologs:

Illumina’s Off-Line Basecaller V1.8 software was used for base calling and demultiplexing. Reads from each species were assembled using Velvet software (v.1.013) (Zerbino and Birney 2008). The number of assembled contigs of one accession from each of the six species with sequence length greater than 400 bp and 1,000 bp ranged from 18,971 to 27,057 and 1,589 to 3,550, respectively. Resulting scaffolds from each species were blasted to each of the other perennial species using reciprocal BLAST at an E-value threshold of e^-20^ and a total of 295 cDNA sequence scaffolds (putatively orthologous gene sets) were identified that were present in each of the six species. These scaffolds were subsequently aligned to the Williams 82 soybean whole genome sequence (http://phytozome.jgi.doe.gov/pz/portal.html#!info?alias=Org_Gmax) to identify the corresponding orthologs in *G. max* and *G. soja*.

#### Species-specific primer design, PCR amplification, and sequence analysis:

A total of 295 sets of species-specific PCR primer pairs were designed with an average predicted amplicon length of 1 Kbp using Primer3 (Rozen and Skaletsky 2000). In the case of the annual species, *G. soja* and *G. max*, only one set of 295 primer pairs was selected based upon the Williams 82 soybean genome sequence (Schmutz *et al.* 2010). These primers were used to amplify genomic DNA from one or two genotypes of each species. The resulting PCR products were run on a 2% agarose gel to determine if a single amplicon was produced. In those cases when a single amplicon appeared to be produced, the resulting PCR product was sequenced on an ABI3130xL (Applied Biosystems, Foster City, CA). To verify that a single amplicon was present, the resulting sequence was analyzed using the DNA analysis software Phred (Ewing *et al.* 1998) and Phrap (http://www.phrap.org/) and the resulting alignments and trace data were visually inspected in the Consed viewer to distinguish, as suggested by Choi *et al.* (2007), those amplicons that were locus specific and those that apparently resulted from amplification of two or more paralogous loci. Finally, of the 295 primer sets designed to putatively orthologous genes, 76 sets that produced a single amplicon in at least six of the eight species were selected (Supplementary File 2) and used for the amplification of genomic DNA for the 10 to 15 accessions from each of the eight species. The resulting amplicons were run on agarose gels and the concentrations were visually compared. Based on these relative concentrations, approximately equimolar amounts of the amplicons from the same accession were combined and the concentration of the combined DNA was determined using the Agilent 2100 Bioanalyzer. The DNA pool of the amplicons from each accession was prepared for sequence analysis on the Illumina HiSeq 2000 to obtain 150 bp single-end reads following the protocol described above, except, 96 sets of indexing adaptors were used for multiplex sequencing which were selected from the adaptors reported by Craig *et al.* (2008) and Hamady *et al.* (2008) and modified to allow paired-end sequence analysis using the Illumina HiSeq 2000. The resulting paired-end reads were de-multiplexed using CASAVA (v. 1.82) and assembled using Velvet software.

#### SNP and indel identification and the estimation of nucleotide diversity:

The scaffolds of each accession were clustered using the 76 gene DNA sequences of their respective species obtained by Sanger sequencing on an ABI3130xL using USEARCH (Edgar 2010). The clustered sequences were aligned using MUSCLE with the gap open penalty of -400, gap extension penalty of 0, center parameter of 0, number of iterations of 16 and distance measure for initial iterations of Kmer4-6 (Edgar 2004). If any portion of an amplicon was missing from an accession, PCR was done using the species-specific primers and the PCR product was sequenced on an ABI3130xL. The sequence was analyzed using Phred and Phrap, after which the sequences were combined with the previously clustered scaffold sequences for SNP and indel detection. Nucleotide diversity, *θ* (Watterson 1975) and *π* (Tajima 1983), of each species was calculated to estimate the genetic diversity of the eight species.

### Phylogenetic analysis

#### RNA extraction, cDNA preparation, and sample preparation for sequence analysis:

A single seed from 10 to 12 accessions of the eight *Glycine* species was scarified and germinated on wet paper in a petri-dish for 3 to 7 days. The germinated seedling was transferred to a paper wick in a plastic pouch filled with nutrient solution (Barker *et al.* 2006) and grown for 3 to 14 days. Total RNA was extracted from each seedling of 10 to 12 accessions for each of the eight *Glycine* species from which poly-A^+^ RNA was isolated. cDNA libraries were constructed following the protocol “Preparing samples for sequencing of mRNA” from Illumina Inc. and prepared using the 96 sets of indexing adaptors following the protocol described above, except, DNA fragments ranging from 250 to 450 bp were isolated. The prepared cDNA libraries from the whole seedlings of the accessions of the same species were normalized, combined and run in a single lane on the Illumina HiSeq 2000 to obtain 150 bp paired-end sequence reads.

#### Sequence analysis and the selection of orthologs:

The number of assembled contigs for the 10 to 15 accessions of each of the eight species with a length greater than 400 bp ranged from 18,070 to 47,579. A total of 153,106 *Phaseolus vulgaris* L. and 189,593 *Vigna unguiculata* (L.) Walp ESTs were downloaded from NCBI and of these, 90,364 of the *P. vulgaris* and 125,808 of the *V. unguiculata* ESTs with sequence length greater than 400 bp were used for the selection of orthologs and used as out-groups in the phylogenetic analysis of the *Glycine* species. To identify orthologs among the eight *Glycine* species and the two out-groups, cDNA sequences from the 95 *Glycine* accessions and ESTs of *P. vulgaris* and *V. unguiculata* were analyzed at an e-value threshold of e^-10^ using Proteinortho v 5.11 (Lechner *et al.* 2011). Proteinortho identifies a single ortholog as well as co-orthologs in those cases where there was more than one gene with very similar sequences (paralogs) found in an accession. If there were co-orthologs present in any accession of the eight species, the entire orthologous set was eliminated. In addition, it was difficult to obtain orthologous genes present in all 95 accessions, so if the ortholog was present in greater than three accessions in each species, the gene was selected for the analysis. The resulting number of selected orthologous gene sets was 52 which were present in 77 of the 95 accessions of the eight *Glycine* species. To verify that the same homeologous gene sets were being compared for the phylogenetic analysis, evolutionary divergence analysis among the species and test for departure from neutrality, the selected 52 gene sets from each of the perennial species were aligned against the whole transcriptome sequences of the corresponding species using BLAST at an e-value threshold of e^-20^. The results showed that the second best alignment score of the 52 gene sets across the six species averaged only 19.8% of the best alignment score and the highest of the secondary alignment scores in any of the six perennial species was 82.5%. In addition, in the case of *G. max* and *G. soja*, we examined the potential homeologs of the 52 genes in *G. max* based on alignment to the whole genome sequence of *G. max* in an effort to further identify paralogous sequences. The mean alignment score of the 2^nd^ best alignment averaged only 26.9% of the best alignment score. The best of the 52 secondary alignment scores was only 76% of the corresponding first alignment score. This was further indication that the 52 genes, very likely, have only one copy in the genome. Therefore, the 52 orthologous gene sets were used for the phylogenetic analysis, the evolutionary divergence analysis among the species and the test for departure from neutrality.

For a separate phylogenetic analysis of *G. canescens*, the assembled contigs and scaffolds of 11 accessions were used. This included the nine accessions used for the phylogenetic analysis of the eight *Glycine* species and two additional accessions. ESTs of the two out-groups, *P. vulgaris* and *V. unguiculata*, with a length greater than 400 bp were also chosen. Proteinortho was used to identify orthologs among the 11 *G. canescens* accessions. A total of 367 gene sets that did not include any of the 52 genes used in the analysis of the eight *Glycine* species were present in at least 7 of the 11 accessions and were used to estimate the relationships among the *G. canescens* accessions.

#### Phylogenetic analysis procedures:

The 52 orthologous gene sets identified in the 77 accessions of the eight *Glycine* species and the 367 gene sets of the 11 *G. canescens* accessions were analyzed using MUSCLE (Edgar 2004) and concatenated using linux commands. Concatenated sequences were analyzed with 1,624 models using jModelTest 2.1.3 (Darriba *et al.* 2012) to select the best nucleotide substitution model. The 1,624 models included 203 different partitions of the Generalized time-reversible (GTR) rate matrix combined with rate variation (+I, +G, +I+G) and equal/unequal base frequencies. Likeliscore for the number of models for the best-fit models within the full set of 1,624 models was calculated and optimized at most 288 models while maintaining model selection accuracy. The best model selected for the orthologs of the *Glycine* species and the 11 G. *canescens* accessions was the same (TPM1uf+I+G). Best models selected by jModelTest 2.1.3. were used in Phyml v3.0 (Guindon *et al.* 2010) with 100 bootstrap replicates. Phylogenetic relationships of the *G. canescens* accessions were used to produce a phylogeography of the accessions using GenGIS (Parks *et al.* 2013). Digital map data were provided by the ORNL DAAC spatial data access tool (http://webmap.ornl.gov/).

### Analysis of evolutionary divergence of genic sequences and identification of trans-specific polymorphisms among Glycine species

Evolutionary divergence among *Glycine* species based on the aligned sequence of 52 genes was estimated using the Tajima-Nei distance method assuming equal substitution rates among sites and between transitional and transversional substitutions (Tajima and Nei 1984).

The distance matrix was calculated using software Mega 7 (Kumar *et al.* 2016). Trans-specific polymorphisms among species were also determined based on the aligned sequence of 52 genes using software Mega 7. In order to test the correlation between the levels of within- and between-population DNA variation as predicted by the neutrality, equilibrium and independence and to test heterogeneity in levels of polymorphism relative to divergence (Haddrill *et al.* 2005), HKA tests (Hudson *et al.* 1987) of the genes between *G. max* and each of other *Glycine* species were performed using the DNASP Beta Version: 6.12.01 downloaded at http://www.ub.edu/dnasp/.

### Data Availability

S1 file contains information of the accessions of annual and perennial Glycine species used for this study. S2 file contains the annual soybean ortholog from each of the 76 orthologous gene sets used in this study. S3 contains HKA test for 52 genes in each species. S4 contains sequence alignment of 52 genes. Supplemental material available at FigShare: https://doi.org/10.25387/g3.7627148.

## Results

### Genetic diversity of the Glycine species

**Figure 2:**
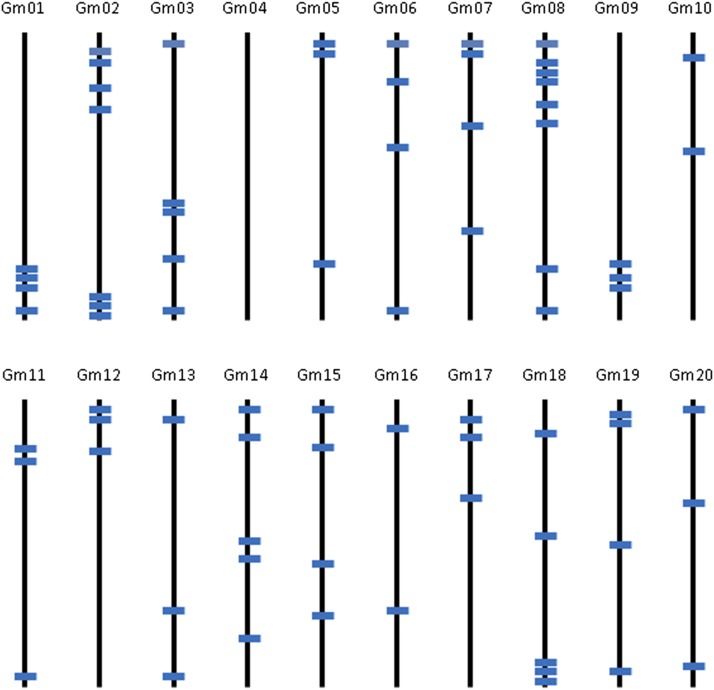
The locations of 76 orthologous genes on the 20 *G. max* chromosomes (Gm1- Gm20). The bars indicate the positions of the 76 genes used for the genetic diversity analysis.

A total of 76 gene sets were selected for the estimation of genetic diversity in the *Glycine* species. The number of fragments amplified in 10 to 15 accessions of each species that produced a single amplicon varied from 63 to 74 and the total length of sequence analyzed ranged from 37.9 to 45.0 Kbp in the eight species (Table 1). Nucleotide diversity measured as *θ* of the two annual species was 0.0011 and 0.0022 for *G. max* and *G. soja*, respectively, and that of the perennial species had up to four times higher genetic diversity than *G. max* with the highest nucleotide diversity in *G. canescens* (*θ*=0.0043bp) followed by *G. stenophita*, *G. cyrtoloba*, *G. syndetika*, *G. falcata* and *G. tomentella* D3 (Table 1). An average nucleotide diversity estimated as *π* also showed a similar trend with the diversity measured as *θ*, where *G. canescens* had the highest diversity (*π*=0.0031bp). However, *G. stenophita* and *G. falcata* had relatively low *π* values compared with their *θ* values. Orthologous gene sets used for this study were distributed on 19 of the 20 soybean chromosomes (Figure 2) and included more than 37 Kbp of genic sequence in each of the eight species.

**Table 1:** Nucleotide diversity (*θ* and *π*) of the *Glycine* species based upon the DNA sequence analysis of orthologous genes identified from 76 orthologous gene sets that produced a single amplicon in at least six of the eight *Glycine* species analyzed using species specific PCR primers

Species	Number of individuals	Number of genes	Total length (bp)	Number of SNPs	Nucleotide diversity (*θ*)	Nucleotide diversity (*π)*
*G. max*	15	70	41,179	148	0.0011	0.0009
*G. soja*	12	70	45,044	295	0.0022	0.0016
*G. canescens*	15	74	44,560	626	0.0043	0.0031
*G. cyrtoloba*	12	63	37,931	366	0.0032	0.0029
*G. falcata*	12	65	37,904	307	0.0027	0.0016
*G. stenophita*	12	71	41,276	406	0.0033	0.0019
*G. syndetika*	10	69	42,120	365	0.0031	0.0025
*G. tomentella* D3	12	67	41,684	202	0.0016	0.0010

### Phylogenetic analysis of the Glycine species and G. canescens

**Figure 3:**
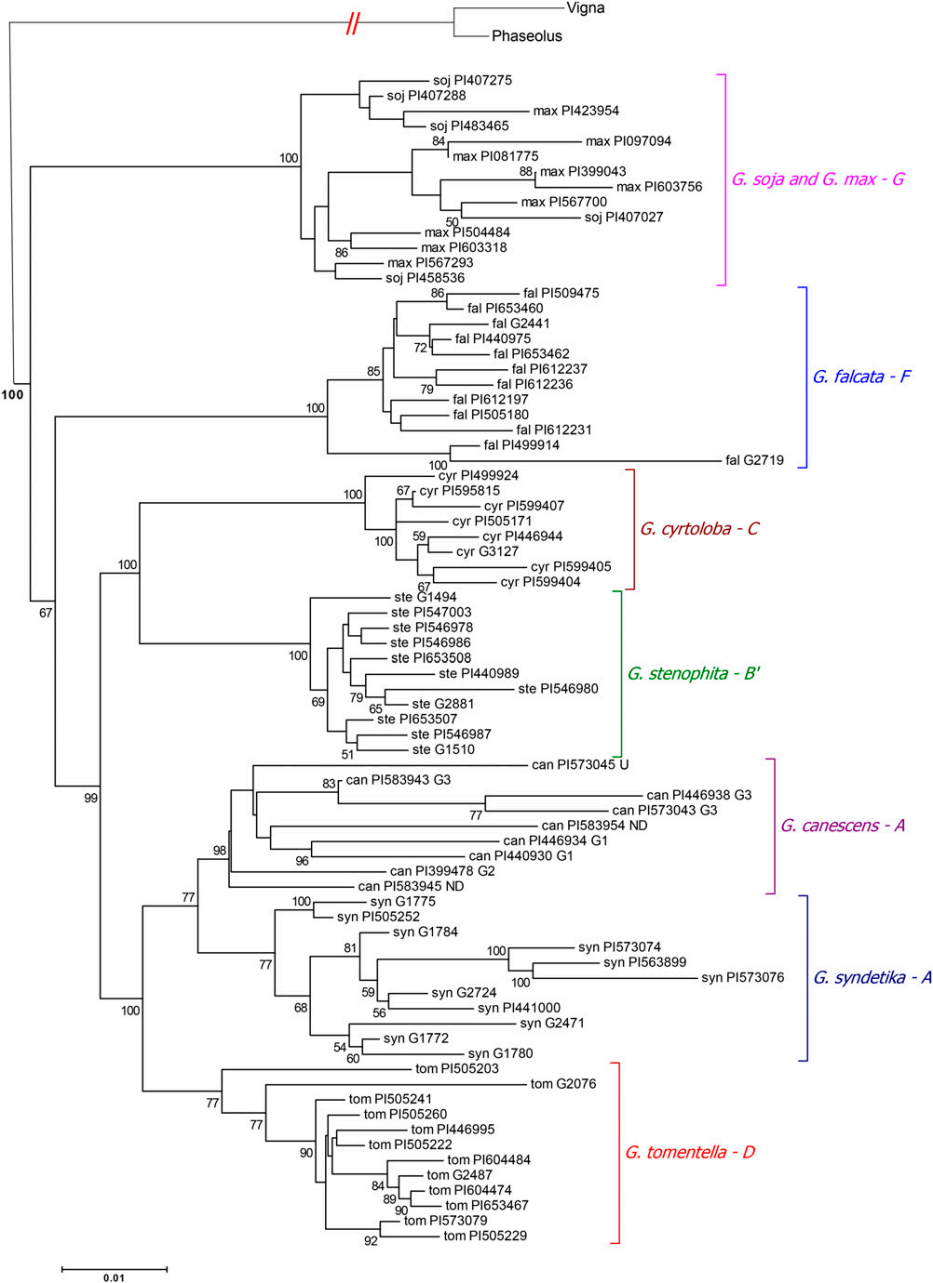
Phylogenetic trees of the *Glycine* species. The phylogenic tree of the 77 accessions of the eight *Glycine* species is based upon 52 orthologous gene sets. The letter next to each species indicates its nuclear genome designation as defined by Singh and Hymowitz (1985a), Hymowitz *et al.* (1998), and Doyle *et al.* (2004). The designation following the PI numbers of the *G. canescens* accessions indicates the subgroup designation (G1 through G3 and U = unassigned to a subgroup) assigned by Brown *et al.* (1990). *G. canescens* accessions followed by “ND” were not included in the Brown *et al.* (1990) study and thus, their subgroup was not determined. Nodes are based on 100 bootstrap replicates. The bootstrap values lower than 50 were eliminated. *Phaseolus vulgaris* L. and *Vigna unguiculata* (L.) Walp are used as out-groups.

A total of 52 orthologous gene sets present in 77 accessions of the eight *Glycine* species and two out-groups, *Phaseolus vulgaris* L. and *Vigna unguiculata* (L.) Walp, produced a concatenated data set approximately 87.6 Kbp in length. Phylogenetic analysis produced a phylogenetic tree (Figure 3) whose topology was quite similar to the genome grouping based upon Histone H3-D (Doyle *et al.* 1996; Sherman-Broyles *et al.* 2014), except *G. cyrtoloba* and *G. stenophita* formed a sister clade in this study. Annuals, *G. soja* and *G. max* of the G genome group, were strongly supported as a monophyletic group sister to the perennials. Within the perennial species, *G. falcata* was a sister of all the other perennial species with a bootstrap value of 67. As expected, the two A group species, *G. canescens* and *G. syndetika*, formed a sister clade which was a sister of the D group species, *G. tomentella* with a bootstrap value of 100. *G. cyrtoloba* of the C group and *G. stenophita* of the B’ group formed a clade which was a sister of the clade including the two A group species, *G. canescens* and *G. syndetika*, and the D group species, *G. tomentella*. The relationships of the clades were supported with moderate and high bootstrap values.

**Figure 4:**
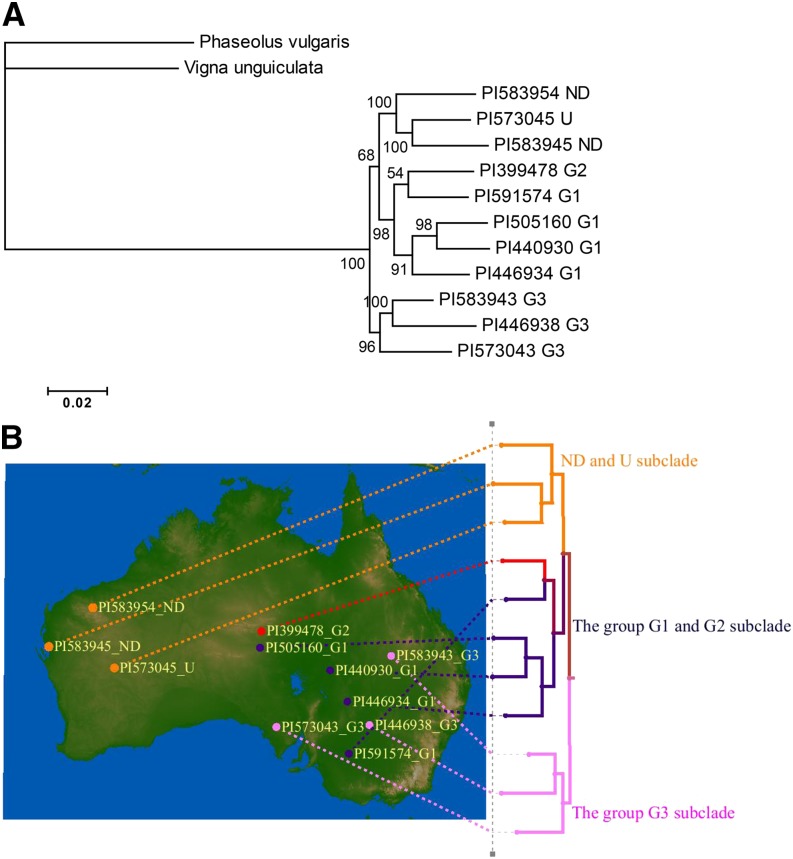
Phylogenetic tree and phylogeography of *G. canescens*. A) The phylogenetic tree is based upon 367 gene sets in the 11 accessions. The letter next to the PI numbers indicates the subgroup designation assigned by Brown *et al.* (1990). *G. canescens* accessions followed by “ND” were not included in the Brown *et al.* (1990) study and thus, their subgroup was not determined. Nodes are based on 100 bootstrap replicates. *Phaseolus vulgaris* L. and *Vigna unguiculata* (L.) Walp are used as out-groups. B) The geographicorigin of each accession of the phylogeny is indicated as a dot on the map and the accessions defined as the same genome group by Brown *et al.* (1990) are assigned the same color. The PI number and the genome group of each accession is indicated next to the dot and the three subclades are indicated.

Of the perennial *Glycine* species, *G. canescens* was the only species whose genetic structure was estimated based on isozyme variation by Brown *et al.* (1990). Thus, the PI number of each *G. canescens* accession is followed by the group (G1, G2, G3, and U: the heterogeneous, ungrouped accession) as assigned by Brown *et al.* (1990) (Figure 3 and Figure 4). In Figure 3, the relationships among the nine *G. canescens* accessions showed that there were two clades, one with the group G3 accessions and the other with the group G1 and one of ND accessions, which were sisters of the group G2, U and one of ND accessions. To verify the relationships among the *G. canescens* accessions in Figure 3, we performed a separate phylogenetic analysis using 367 orthologous gene sets identified in the 11 *G. canescens* accessions and the two out-groups which produced concatenated sequences with a length of 462 Kbp. Results showed that there were two major clades, one included accessions from the genome group G3 and the other included the remainder of the accessions. The latter clade included two clades, the first with the U and ND accessions and the second with the group G1 accessions, as well as the single G2 accession (Figure 4A). In addition, the phylogeography of the *G. canescens* accessions showed that the distance between the geographic origins of the accessions increased as their phylogenic relationships were more distant (Figure 4B).

### Divergence among species

The Tajima-Nei distance analysis based on the 52 gene sequences (Supplementary File 3) showed that the divergence between *G. max* and *G. soja* is the smallest (0.009), perennial species *G. falcata* was the most divergent from *G. max*, followed by *G. cyrtoloba*, *G. syndetika*, *G. tomentella* D3, *G. stenophita* and *G. canescens*. Among the perennials, the largest differentiation was observed between *G. falcata* and *G. cyrtoloba*, and between *G. falcata* and *G. syndetika*. while the smallest differentiation was observed between *G. canescens and G. syndetika*. The divergence among the two *Glycine* species in group A was small (Table 2).

**Table 2:** Estimates of evolutionary divergence among *Glycine* species based on DNA sequence analyses of 52 orthologous genes that produced a single amplicon in at least six of the eight *Glycine* species analyzed using species specific PCR primers

	*G. max*	*G. soja*	*G. canescens*	*G. cyrtoloba*	*G. falcata*	*G. stenophita*	*G. syndetika*
*G.max*	0						
*G. soja*	0.009	0					
*G. canescens*	0.042	0.043	0				
*G. cyrtoloba*	0.050	0.049	0.041	0			
*G. falcata*	0.052	0.048	0.045	0.047	0		
*G. stenophita*	0.042	0.042	0.032	0.037	0.045	0	
*G. syndetika*	0.046	0.044	0.019	0.046	0.047	0.036	0
*G. tomentella D3*	0.043	0.041	0.025	0.041	0.042	0.032	0.028

### Trans-specific polymorphism among Glycine species

**Figure 5:**
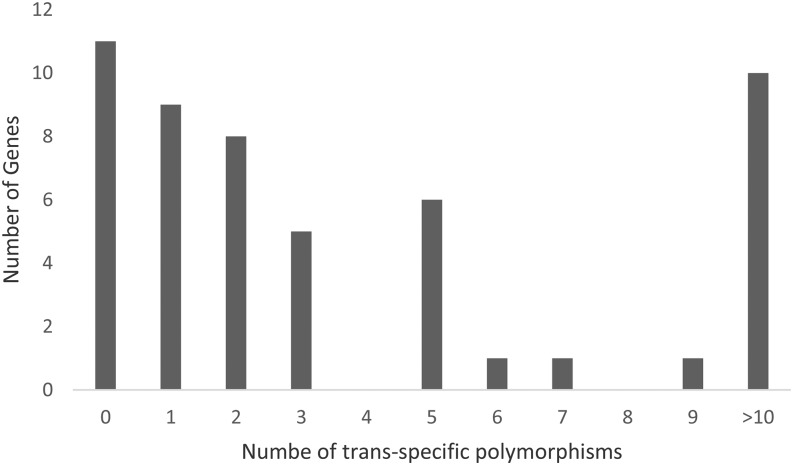
Distribution of genes with different number of *trans*-specific polymorphisms.

Of the 52 genes, 41 genes contained 1 to 40 *trans*-specific polymorphic loci. There were ten genes with at least 10 *trans*-specific polymorphisms (Figure 5). Among the 316 *trans*-specific polymorphic loci, a total of 152, 81, 61 and 22 were A/G (T/C), A/C (T/G), A/T (T/A), and C/G (G/C), respectively. The transitions (*i.e.*, C/T or A/G) occurred more often than any of the transversions A/C, A/T or C/G.

Of the 316 *trans*-specific polymorphic loci, a total of 223, 66, 9, 14, and 4 were *trans*-specific among 2, 3, 4, 5 or 6 *Glycine* species, respectively. Further analysis showed that *G. max* *vs.* *G. syndetika* had the highest number of *trans*-specific polymorphic loci, followed by *G. syndetika vs. G. tomentella*, *G. falcata* *vs.* *G. syndetika*, *G. soja vs. G. syndetika*, *and G. canescens vs. G. max*. Interestingly, *G. syndetika* had the highest but *G. cyrtoloba* had the lowest number of *trans*-specific polymorphisms with other species (Table 3). The *trans*-specific polymorphic loci were more likely observed between the species that were less divergent from each other.

**Table 3:** Number of *trans*-specific polymorphisms between *Glycine* species

Species	Number of *trans*-specific polymorphisms
*G. max/G. syndetika*	59
*G. syndetika/G. tomentella*	47
*G. falcata/G. syndetika*	44
*G. soja/G. syndetika*	41
*G. canescens/G. max*	37
*G. falcata/G. soja*	36
*G. canescens/G. falcata*	34
*G. canescens/G. tomentella*	34
*G. canescens/G. syndetika*	33
*G. max/G. soja*	32
*G. falcata/G. tomentella*	30
*G. falcata/G. max*	29
*G. max/G. tomentella*	25
*G. soja/G. tomentella*	25
*G. stenophita/G. syndetika*	24
*G. max/G. stenophita*	20
*G. canescens/G. soja*	19
*G. soja/G. stenophita*	17
*G. stenophita/G. tomentella*	16
*G. falcata/G. stenophita*	15
*G. cyrtoloba/G. falcata*	11
*G. cyrtoloba/G. max*	11
*G. cyrtoloba/G. syndetika*	11
*G. canescens/G. cyrtoloba*	10
*G. canescens/G. stenophita*	7
*G. cyrtoloba/G. tomentella*	5
*G. cyrtoloba/G. soja*	1
*G. cyrtoloba/G. stenophita*	1
*G. falcata/G. cyrtoloba*	1

### HKA test of genes under evolution selection between G. max and other Glycine species

Because of missing genic sequence and lack of sequence polymorphism in some accessions, HKA values between *G. max* and other species were obtained for 40 of the 52 genes. Of the 169 HKA values acquired between *G. max* and some other *Glycine* species, only 6 (3.6%) from 15 genes were deviated from neutral evolution expectation at *P* = 0.05. The number of genes with significant HKA values between *G. max* and each of other species varied from 1 to 2 at *P* = 0.05 (Supplementary File 3).

## Discussion

### Nucleotide diversity

The sets of *Glycine* accessions used in this study were selected to represent the geographical range of the origins of the respective species for the purpose of obtaining a representative sampling in order to provide reliable estimates of genetic diversity. An average genetic diversity of *G. soja* was twice that of *G. max* which was similar to the previous report (Hyten *et al.* 2006). The values of nucleotide diversity reported by Hyten *et al.* (2006) were *θ*=0.00235 for 26 *G. soja* accessions collected from China, Korea, Taiwan, Russia and Japan and *θ=* 0.00099 for a set of *G. max* genotypes consisting of 52 Asian landraces, 17 N. American ancestral cultivars and 25 N. American elite cultivars released between 1977 and 1990. Very similar nucleotide diversity values for *G. max* of *θ*=0.00097 and *θ*=0.00099 were reported by Zhu *et al.* (2003) and Choi *et al.* (2007), respectively. Of all the perennials, *G. canescens* has the most extensive geographic distribution across Australia (Brown *et al.* 1990; González-Orozco *et al.* 2012), mostly in very dry areas (Figure 1), while the other perennial species have more localized ranges. As might be expected by its broad geographical distribution and the presence of at least three subgroups within the species as reported by Brown *et al.* (1990), *G. canescens* had the highest nucleotide diversity of the *Glycine* species which was approximately four times higher than that of *G. max* as estimated by *θ*. With the exception of *G. tomentella* D3 which had unexpectedly low nucleotide diversity (*θ*=0.0016), the other perennial species, *G. cyrtoloba*, *G. falcata*, *G. stenophita*, and *G. syndetika*, had a mean nucleotide diversity (*θ*) higher than that of the annual *G. soja* (*θ*=0.0022) (Table 1). However, the average diversity of the *Glycine* species estimated by *π* was not in complete agreement with the *θ* values. *G. canescens* also showed the highest diversity estimated as *π*, whereas *G. falcata* and *G. stenophita* had a relatively low nucleotide diversity (*π*) as compared with their *θ* values.

There has been no extensive genetic diversity analysis reported on the perennial *Glycine* species because of a lack of nucleotide sequence availability, so it is difficult to compare the current estimation of genetic diversity with other studies. However, we used a total of 63 to 74 orthologous gene sets in the annual and perennial *Glycine* species that were distributed on 19 of the 20 soybean chromosomes (Figure 2). This is the first report to provide an estimate of genetic diversity of the *Glycine* species based upon sequences distributed across the genomes of the annual species and given the close relationship of the annual and perennial species, these sequences are also very likely to be spread across the genomes of the perennial species.

### Phylogenetic analysis

An important step after the collection of new germplasm is to determine its relationship with other known species by comparing morphological traits, by cytological and crossing studies and/or by the analysis of molecular variability. The early phylogenetic analyses of the perennial *Glycine* species were conducted mostly using interspecies crossability and cytogenetic studies with the ultimate intention of the discovery and introgression of beneficial genes/alleles into cultivated soybeans. These efforts resulted in the construction of the first nuclear genome designation of the genus *Glycine* composed of six diploid genome groups (Singh and Hymowitz 1985a), which ultimately included nine genome groups from A to I (Hymowitz *et al.* 1998) consisting of 25 perennial species (Doyle *et al.* 2004; Sherman-Broyles *et al.* 2014). Based upon these previous analyses, the eight *Glycine* species used in the current study belong to six of the nine genome groups. The two annual *Glycine* species, *G. soja* and *G. max*, belong to the G genome group; the perennial species *G. canescens* and *G. syndetika* belong to the A group; *G. stenophita* to the group B’ which was integrated into the genome grouping system by Brown *et al.* (2002); *G. cyrtoloba* to the C group; *G. tomentella* D3 to the D group, and *G. falcata* to the F group. There have been studies reporting phylogenetic relationships of the *Glycine* species based on nucleotide sequence variation in a single or small number of nuclear DNA sequences (Kollipara *et al.* 1997; Doyle *et al.* 1999a; Brown *et al.* 2002) or chloroplast DNA sequence (Doyle *et al.* 1990; Sakai *et al.* 2003). As might be expected, the tree estimations from different studies were different (Doyle *et al.* 2003b) because different genes had a different gene history which might not represent the history of the taxa (Rokas *et al.* 2003). In this study, we used approximately 87.6 Kbp of common sequence from 52 orthologous gene sets present in the 77 accessions of the eight *Glycine* species and two out-groups, *Phaseolus vulgaris* L. and *Vigna unguiculata* (L.) Walp, for the estimation of the phylogenetic relationships. This analysis should provide a reasonable estimation of the phylogenetic relationships of the eight *Glycine* species.

The phylogenetic relationships estimated in this study agreed with a sister relationship between the monophyletic annual and perennial subgenera reported in the previous studies (Doyle *et al.* 2003a) and it agreed with the relationships based upon Histone H3-D (Sherman-Broyles *et al.* 2014), except that *G. cyrtoloba* of the C genome group and *G. stenophita* of the B’ group formed a sister clade (Figure 3). The close relationship between *G. cyrtoloba* and the B genome group was suggested by Singh (2016). Based upon a cytogenetic analysis, Singh also reported that the D genome group had evolved from the A group which agreed with the phylogenetic analysis performed in this study where the A group species, *G. canescens* and *G. syndetika* and the D group species, *G. tomentella*, formed a sister clade (Figure 3).

Of the six perennial species, *G. canescens* is the species with the most geographically widespread distribution in Australia (Brown *et al.* 1990; González-Orozco *et al.* 2012). Brown *et al.* (1990) estimated the genetic structure of *G. canescens* using the variation of 11 isozymes and reported that *G. canescens* could be classified into five groups, the groups G1, G2, and G3 which had different isozyme variation patterns, the U group which did not belong to any of the other groups but shared more alleles with group G3 and the X group which was morphologically similar to *G. clandestina*. In addition, there were *G. canescens* accessions collected more recently whose genome groups have not been determined. Two of the *G. canescens* accessions used in this study belong in this latter category. In Figure 3 the clade of *G. canescens* includes only 9 accessions because a number of the 52 orthologous gene sets used in the analysis were not present in the two *G. canescens* accessions PI505160 and PI591574. To confirm the relationships among the 9 *G. canescens* accessions we performed a separate phylogenetic analysis using 367 orthologous gene sets in 11 accessions which added two more group G1 accessions to the analysis. In both analyses the genome group G1 accessions were clustered in as single clade as were the group G3 accessions (Figure 3 and Figure 4A). In Figure 4B, the group G1 accessions originated from the Northern Territory, South Australia, New South Wales, and Victoria, however, their geographic origins were limited to longitudes between 134°E and 143°E and latitudes between 25°S and 35°S where the maximum physical distance among the geographical origins of the four G1 accessions was 1,270 Km followed by 946 Km and 684 Km (Figure 4B). The group G3 accessions were from New South Wales, South Australia, and Queensland where the average precipitation is greater than the areas from which the other accessions originated (Figure 1) and as was the case for the group G1 accessions. As the distance between the geographic origins of the accessions increased their phylogenic relationships were more distant. (Figure 4B). The two ND and U accessions were in a distinct clade in the phylogenetic tree using the 367 gene sets (Figure 4A) whereas one of ND accessions was clustered with the group G1 accessions in the tree using the 52 gene sets (Figure 3). The ND and U accessions originated from Western Australia which is geographically apart from the regions where the group G1, G2, and G3 accessions originated (Figure 4B). Based on the geographic origins of the ND and U accessions, the clustering using the 367 gene sets would appear to be more reliable. In this study, we used only one group G2 accession from the Northern Territory so that we do not know the relationships among the group G2 accessions. However, it was assumed that the genome group G2 was more closely related to the group G1 than the group G3 based upon the phylogenetic tree in Figure 4A. Based on the discussion above, in the case of *G. canescens*, the phylogenetic analysis using the 367 gene sets was more reliable and agreed with the groupings as reported by Brown *et al.* (1990). Clearly, *G. canescens* appeared to be the most diverse of the perennial species examined in this study based upon sequence diversity, geographical origins and the phylogenetic analysis.

This study is the first to provide an estimate of genetic diversity of the perennial *Glycine* species and we found that all the perennial species had higher genetic diversity (*θ* and *π*) than the annual species, except *G. tomentella* D3 and *G. falcata*, based upon the analysis of a total of 63 - 74 orthologous gene sets. The greater genetic diversity of most of the perennial *Glycine* offers the hope of variation for genetically controlled traits that are not present in the annual *Glycine* species. Some of the perennial *Glycine* have been adapted to the environments where the average annual precipitation on the Australian continent was approximately 450 mm for the last 100 years (http://www.abs.gov.au). The regions where *G. canescens* are found are the driest and hottest areas in Australia with an annual precipitation of less than 300 mm and an average daily maximum temperature of 33°C in January. All of the other perennial *Glycine* are geographically localized from the northeastern to southeastern coastal areas in Australia (Figure 1) where the average maximum temperature in January is 27°C with relatively higher annual precipitation ranging from 500 to 1600 mm. Therefore, we can assume that the perennial *Glycine*, and particularly *G. canescens* with its origin in the driest and warmest regions of Australia, could have numerous stress related genes/alleles to survive in drought and high temperatures. The genes/alleles would associate with candidate traits that need to be integrated into cultivated soybean to cope with future climate change. Given the likelihood of climate change, it seems likely that genes controlling resistance to heat and drought will be of particular importance in future soybean cultivars. Given the rapidity with which high density genetic maps can now be developed using genotyping by sequencing (Elshire *et al.* 2011), gene identification and cloning from the perennial *Glycine* species may become a relatively rapid process that can be used as a source of genes/alleles in cultivated soybean.

### Divergence analysis among Glycine species

Among the perennial species, the three species *G. tomentella*, *G. canescens*, *and G. syndetika* were less divergent from each other, but were the most divergent from *G. falcata* based on the Tajima-Nei distance analysis method. The results were consistent with those based on other models such as p-distance (Nei and Kumar 2000), Jukes-Cantor distance (Jukes and Cantor 1969), the Kimura 2-parameter distance (Kimura 1980) and the Tamura-Nei distance (Tamura and Nei 1993). The relationship among *Glycine* species based on sequence divergence was also consistent with that inferred by the phylogenic tree (Figure 3) and the outcome of hybridization among *Glycine* species. For example, we observed that *G. max* was the most divergent from *G. falcata*, followed by *G. cyrtoloba*, *G. syndetika*, *G. tomentella* D3, *G. stenophita* and *G. canescens*. This conclusion was congruent with the crossability report of soybean cultivars ‘Lincoln’ and ‘Hark’ with *G. tomentella*, *G. canescens*, *G. falcata*, *G. tabacina*, *G. latrobeana*, *G. clandestina*, *and G. latifolia*, respectively (Broué *et al.* 1982). From those crosses, hybrid seeds were only obtained from the crosses with *G. tomentella* and *G. canescens*. Successful hybridization of cultivated soybean with *G. tomentella* and *G. canescens* was also reported in a number of other studies (Newell *et al.* 1987; Newell and Hymowitz 1982; Singh and Hymowitz 1985b; Bodanese-Zanettini *et al.* 1996). Besides cytogenetic differences between *G. falcata* and other *Glycine species* reported by Putievsky and Broue (1979), the pod production habit of *G. falcata* was also different, *G. falcata* is the only species developing underground pods. The relationship among *Glycine* species and the estimates of divergence between soybean and other *Glycine* species will facilitate the selection of perennials that will most likely be successful to hybridize with cultivated soybean.

### Trans-specific mutations

The higher occurrence of transitions (*i.e.*, C/T or A/G) than transversions was also described by Brown *et al.* (1982) and Keller *et al.* (2007). Transition mutations are more easily generated due to molecular shape, and are less likely to be removed by natural selection because they more often create synonymous amino acids as demonstrated in influenza virus and HIV by Lyons and Lauring (2017). Although *trans*-specific polymorphic loci were more frequent between closely related species, we didn’t observe a high number of those loci between *G. max* and *G. soja* due to allele fixation in 52 genic sequences in *Glycine soja* and *G. max*. We observed that *G. max* shared the highest number of *trans*-specific polymorphism with *G. syndetika*, followed by *G. canescens*. Previous studies classified *G. syndetika* and *G. canescens* into genome group A based on interspecific crossability, meiotic chromosome pairing or seed protein electrophoresis (Hymowitz *et al.* 1998; Sherman‐Broyles *et al.* 2014; Singh and Hymowitz 1985a) and the two species were in the sister clade of *G. tomentella* (D genome group) (Doyle *et al.* 2003a; Sherman‐Broyles *et al.* 2014). So far, no attempt has been made to cross *G. syndetika* with *G. max*, the crossability of *G. tomentella and G. canescens* with *G. max* suggests that the *G. syndetika* is not divergent from *G. max*.

A number of studies have reported the relationship of a small number of *Glycine* species (Kollipara *et al.* 1997; Doyle *et al.* 1990; Doyle *et al.* 1999a; Sherman-Broyles *et al.* 2014), and conclusions from those studies generally varied depending on taxon sampling and the locus that was used (Sherman-Broyles *et al.* 2014). Sherman-Broyles *et al.* (2014) analyzed pair-wise distance among *Glycine* species based on SNPs in plastomes and concluded that the distance of *G. max* with *G. syndetika* was smaller than that of *G. max* with *G. falcate* or *G. cyrtoloba*. In another study, the divergence between *G. soja* and *G. canescen* was smaller than that between *G. soja* and *G. falcate*, or between *G. soja* and *G. cyrtoloba* based on restriction-endonuclease site variation of chloroplast DNA (Doyle *et al.* 1990). Because species topology may change according to the order of the species arranged on the phylogenic tree, topology sometimes may not represent the true divergence among species, divergence estimates by distance measures may be more reliable.

### Neutral evolution between G. max and other Glycine species

Approximately 4% of the genes between *G. max* and other *Glycine* species deviated from neutral evolution based on the HKA test. The percentage was lower than that reported between *Drosophila simulans and Drosophila meleanogaster* and between humans and monkeys, *e.g.*, approximately 50% of the amino acid substitutions within genes between *Drosophila simulans and Drosophila meleanogaster* were driven by positive selection (Brookfield and Sharp 1994; Sawyer and Hartl 1992), and approximately 35% between humans and old-world monkeys experienced adaptive evolution (Fay *et al.* 2001). Our data showed that most gene sequences were homogeneous in levels of polymorphism and divergence between *G. max* and other *Glycine* species, indicating that all the *Glycine* perennial species may have experienced very similar evolutionary selection.
